# Clinical and metabolic characteristics of endometrial lesions in polycystic ovary syndrome at reproductive age

**DOI:** 10.1186/s12905-023-02339-7

**Published:** 2023-05-06

**Authors:** Xiaozhu Zhong⋅, Yang Li⋅, Weiying Liang, Qiyue Hu, Anqi Zeng, Miao Ding, Dongmei Chen, Meiqing Xie

**Affiliations:** Department of Obstetrics and Gynecology, Sun Yat-sen Memorial Hospital, Sun Yat-sen University, Guangzhou, 510120 China

**Keywords:** Polycystic ovary syndrome, Endometrial lesions, Obesity, Metformin, Hormones

## Abstract

**Background:**

We aimed to explore the clinical and metabolic characteristics in polycystic ovary syndrome (PCOS) patients with different endometrial lesions.

**Methods:**

234 PCOS patients who underwent hysteroscopy and endometrial biopsy were categorized into four groups: (1) normal endometrium (control group, n = 98), (2) endometrial polyp (EP group, n = 92), (3) endometrial hyperplasia (EH group, n = 33), (4) endometrial cancer (EC group, n = 11). Serum sex hormone levels, 75 g oral glucose tolerance test, insulin release test, fasting plasma lipid, complete blood count and coagulation parameters were measured and analyzed.

**Results:**

Body mass index and triglyceride level of the EH group were higher while average menstrual cycle length was longer in comparison with the control and EP group. Sex hormone-binding globulin (SHBG) and high density lipoprotein were lower in the EH group than that in the control group. 36% of the patients in the EH group suggested obesity, higher than the other three groups. Using multivariant regression analysis, patients with free androgen index > 5 had higher risk of EH (OR 5.70; 95% CI 1.05–31.01), while metformin appeared to be a protective factor for EH (OR 0.12; 95% CI 0.02–0.80). Metformin and hormones (oral contraceptives or progestogen) were shown to be protective factors for EP (OR 0.09; 95% CI 0.02–0.42; OR 0.10; 95% CI 0.02–0.56). Hormones therapy appeared to be a protective factor for EC (OR 0.05; 95% CI 0.01–0.39).

**Conclusion:**

Obesity, prolonged menstrual cycle, decreased SHBG, and dyslipidemia are risk factors for EH in patients with PCOS. Oral contraceptives, progestogen and metformin are recommended for prevention and treatment of endometrial lesions in PCOS patients.

## Background

Polycystic ovary syndrome (PCOS) is a prevalent endocrine and metabolic disorder affecting 5–20% of reproductive-aged women [1]. According to the Rotterdam criteria, PCOS is characterized as chronic anovulation, hyperandrogenism, polycystic changes in the ovaries, obesity and insulin resistance. Women with PCOS are at increased risk for glucose intolerance and type 2 diabetes mellitus, hepatic steatosis, hypertension, dyslipidemia, vascular thrombosis, cerebrovascular accidents, subfertility, endometrial carcinoma, ovarian malignancy; and mood and psychosexual disorders [1].

Anovulation, insulin resistance, hyperandrogenism, progesterone resistance or insufficiency and low-grade chronic inflammatory state, can disrupt the endometrium in patients with PCOS and can lead to endometrial hyperplasia and cancer [2]. However, the specific etiology of PCOS associated-endometrial lesions remain to be studied.

We aim to explore whether there are association between clinical, metabolic features of PCOS and endometrial lesions. To the best of our knowledge, this is the first study presenting the different endometrial status in PCOS patients.

## Methods

### Patient information collection

This was a retrospective case-control study. The study was approved by the institutional review board of Sun Yat-Sen Memorial Hospital, Sun Yat-Sen University, Guangzhou, Guangdong, China (Ethical approvement: SYSEC-KY-KS-2022-064). Two hundred and thirty-four women aged from 18 to 45 years old who were admitted to the gynecology department of Sun Yat-sen Memorial Hospital, Sun Yat-sen University between January 2015 and December 2020 for surgery were included.

The following criteria were necessary for participation in the study: (1) aged 18–45 years old; (2) diagnosis of PCOS according to the Rotterdam 2003 criteria [3]. The diagnosis of PCOS requires meeting at least two of the following three criteria: (a) clinical and/or biochemical signs of hyperandrogenism; (b) oligomenorrhea and/or anovulation; (c) polycystic ovaries on pelvic ultrasound examination, and exclusion of other androgen excess diseases, such as hyperprolactinemia, androgen-secreting tumors, non-classic adrenal hyperplasia, Cushing’s syndrome, thyroid dysfunction, etc. The controls were women with PCOS who underwent hysteroscopic surgery for intrauterine adhesion, cesarean scar defect, reproductive malformation, salpingemphraxis or hydrosalpinx within the same period. The diagnosis of endometrial lesions in all patients was based on postoperative pathological examination.

The study group included 92 women with EPs, 33 women with EH, 11 women with EC and the control group included 98 women with normal endometrium. The histopathology of 11 patients with EC was endometrioid adenocarcinoma, of which 90.9% (10/11) were stages I disease and 9.1% (1/11) were stage II diseases.

The demographic and clinical information including menstrual history, height, weight, waist circumference, hip circumference, history of metformin/ hormones (oral contraceptives, progestogen) use, serum lipid levels, complete blood count, coagulation tests and other data of the two groups were collected. Body mass index (BMI) was calculated as the weight (kg) divided by the square of the height (m^2^). According to criteria from the Working Group on Obesity in China (WGOC) [4]: BMI is the normal range in 18.5, BMI more than 24 overweight and obese BMI greater than 28. A 75 g-OGTT (oral glucose tolerance test) and insulin release test were performed before surgery in both groups, and blood glucose and insulin values at different times (0-1-2 h) were obtained. Homeostasis model insulin resistance index (HOMA-IR) was used to evaluate the IR degree and calculated as follows: HOMA-IR = Fasting insulin (mU/L) × Fasting plasma glucose (mmol/L)/22.5. Insulin resistance was diagnosed if one of the following three criteria were met [5]: (1) HOMA-IR ≥ 2.14, (2) Fasting insulin ≥ 15 mu/L, (3) 2-h insulin ≥ 150 mu/L. Impaired fasting glucose was defined as Fasting plasma glucose ≥ 6.1 mmol/L, but < 7 mmol/L, impaired glucose tolerance was defined as 2-h glucose ≥ 7.8 mmol/L, but < 11.1 mmol/L according to the China guideline for diabetes. Free androgen index (FAI) was calculated as follows: (100×testosterone [nmol/L])/ sex hormone binding globulin (SHBG) [nmol/L]). Hyperandrogenism was defined as FAI ≥ 5.

### Laboratory tests

Prolactin (PRL), estradiol (E2), progesterone (P), testosterone, follicle-stimulating hormone (FSH), luteinizing hormone (LH), SHBG, dehydroepiandrosterone (DHEA) and Anti-müllerian hormone (AMH) were measured by chemiluminescence (ACS180 SE, Bayer, Germany). 17α-hydroxyprogesterone was measured by an ELISA Kit (EIA1292, DRG, Germany). Fasting peripheral blood samples were collected during the follicular period at least 12 weeks after the last abortion. Complete blood count was performed by cytological analysis (Sysmex XN9000, Kobe, Japan). Serum lipid levels, including total cholesterol (TC), triglyceride (TG), low density lipoprotein (LDL), high density lipoprotein (HDL) were measured with a biochemical analyzer (POCT workstation, Ottaman; Upper, Shanghai, China). Plasma glucose was measured with a glucose oxidase assay (AU5821; Beckman Coulter, Miami, FL, USA). Insulin was measured by a chemiluminescence immune detection system (ADVIA Centaur XP; Siemens, Beijing, China). Coagulation parameters, including D-dimer and fibrinogen were measured with a coagulation analyzer (CS-5100; Sysmex, Kobe, Japan).

### Statistical analysis

Kolmogorov-Smirnov test was used to verify the normality of continuous variables. Continuous variables were expressed as means ± standard deviations and compared by analysis of variance (ANOVA). Continuous variables with abnormal distributions were expressed as medians (interquartile range) and compared by Kruskal-Wallis H test. Categorical variables were expressed as relative frequencies and compared by chi-squared test. Multivariate logistic regression model (forward stepwise) was used to evaluate the probable risk factors for endometrial lesions. Statistical analyses were performed using SPSS, version 25.0 (SPSS Inc., Chicago, IL, USA). *P* < 0.05 was considered statistically significant.

## Results

The clinical characteristics of PCOS patients with different endometrial status were shown in Table 1. No significant difference was observed in waistline (*P* = 0.686), hipline (*P* = 0.156), waist hip rate (*P* = 0.064), gravidity (*P* = 0.083), parity (*P* = 0.300), prevalence of infertility (*P* = 0.249), hyperandrogenism (*P* = 0.480), insulin resistance (*P* = 0.410), diabetes (*P* = 0.301), impaired fasting glucose (*P* = 0.876) and impaired glucose tolerance (*P* = 0.440).

**Table 1:** Comparison of baseline clinical characteristics of polycystic ovary syndrome patients with different endometrial status

Characteristics	All patients (n = 234)	Normal endometrium(n = 98)	Endometrial polyp (n = 92)	Endometrial hyperplasia (n = 33)	Endometrial cancer (n = 11)	F/H/c2	*P* value
Age(years)	29.47 ± 4.73	28.10 ± 4.69^abc^	29.86 ± 4.43^c^	31.09 ± 4.57	33.55 ± 4.03^a^	7.533	0.000
Body mass index (kg/m^2^)	23.94 ± 4.97	23.46 ± 5.04^b^	23.40 ± 4.17^b^	26.28 ± 6.30^a^	25.29 ± 4.99	2.827	0.040
Obesity	40(17.09)	14(14.29)^b^	11(11.96)^b^	12(36.36)^ac^	3(27.27)^b^	11.533	0.009
Overweight	50(21.37)	23(23.47)	18(19.57)	4(12.12)	5(45.45)	4.702	0.195
Waist hip rate	0.86(0.82–0.89)	0.89(0.85–0.90)	0.84(0.80–0.88)	0.85(0.81–0.87)	0.88(0.83–0.91)	7.277	0.064
Average menstrual cycle(day)	45.00(35.00–75.00)	44.00(33.50–60.00)^b^	45.00(33.50–63.50)^b^	80.00(58.25–105.00)^a^	60.00(40.13–82.50)	18.409	0.000
Prolonged menstruation	40(17.09)	4(4.08)^abc^	18(19.57)^bc^	11(33.33)^ac^	7(63.64)^ab^	35.059	0.000
Menorrhagia	71(30.34)	1(1.02)^abc^	43 (46.74)^c^	18(54.55)^c^	9(81.82)	74.505	0.000
Infertility	129(55.13)	55(56.12)	55(59.78)	13(39.39)	6(54.54)	4.117	0.249
Polycysticovary	209(89.32)	96(97.96)^abc^	76(82.61)^c^	27(81.82)	10(90.90)^a^	13.406	0.004
Hyperandrogenism	139(59.40)	55(56.12)	54(58.69)	25(75.76)	5(45.45)	2.473	0.480
Insulin resistance	124(52.99)	47(47.96)	50(54.35)	22(66.67)	5(45.45)	2.882	0.410
Diabetes	34(14.53)	11(11.22)	13(14.13)	7(21.21)	3(27.27)	3.653	0.301
Impaired fasting glucose	5(2.14)	3(3.06)	1(1.09)	1(3.03)	0	0.690	0.876
Impaired glucose tolerance	46(19.66)	19(19.39)	16(17.39)	7(21.21)	4(36.36)	2.704	0.440
Taking hormones	161(68.8)	91(92.86)^abc^	44(47.83)^b^	22(66.67)^ac^	4(36.36)^b^	36.103	0.000
Taking metformin	61(26.07)	45(45.92)^abc^	8(8.69)^bc^	6(18.18)^a^	2(18.18)^a^	29.229	0.000

*Data are means ± standard or medians (interquartile range) or n (%).* ^*a*^ *compared to endometrial polyp, p < 0.05;* ^*b*^ *compared to endometrial hyperplasia, p < 0.05;* ^*c*^ *compared to endometrial cancer, p < 0.05*

The mean age of patients in the EC group was the highest, and that in the control group was lower than the other three groups (28.1 ± 4.69 years vs. 29.86 ± 4.43, 31.09 ± 4.57, 33.55 ± 4.03 years, *P* = 0.000). The mean BMI of the EH group was higher than the control group and the EP group (26.28 ± 6.30 kg/m^2^ vs. 23.46 ± 5.04, 23.40 ± 4.17 kg/m^2^, *P* = 0.040). The average menstrual cycle length of patients in the EH group was longer than the control group and the EP group [80.00(58.25–105.00) days vs. 44.00(33.50–60.00), 45.00(33.50–63.50) days, *P* = 0.000]. Fewer spontaneous abortion of patients was observed in the EH group compared with the control group [0(0–0) vs. 0(0–2), *P* = 0.013].

Overall, 36.36% of the patients in the EH group suggested obesity, which was higher than the other three groups (14.29%, 11.96% and 27.27%, *P* = 0.000). 63.64% of the patients in the EC group had prolonged menstruation compared to the other three groups (4.08%, 19.57% and 33.33%, *P* = 0.000), and 81.82% had menorrhagia (1.02%, 46.74% and 54.55%, *P* = 0.000). 92.86% and 45.92% of the patients in the control group had taken hormones and metformin, which was higher than the other three groups (47.83%, 66.67% and 36.36%, *P* = 0.000; 8.69%, 18.18% and 18.18%, *P* = 0.000).

**Laboratory parameters among polycystic ovary syndrome patients with different endometrial status were shown in** Table 2. There were no significant differences in levels of PRL, FSH, LH, E2, P, T, 17α hydroxy progesterone, FAI, AMH, GLU0, GLU2h, INS 0, INS2h, HOMA-IR, TSH, TC, LDL, WBC, RBC, prothrombin time,fibrinogen, D-dimer (*P* > 0.05). Level of DHEA was higher in the EP group than in the control group [488.10(231.05-1301.98) ug/dL vs. 283.70(190.40-333.60) ug/dL, *P* = 0.000]. Level of SHBG [25.44(15.05–35.77) nmol/L vs. 44.68(28.82–73.24) nmol/L, *P* = 0.049] and HDL [1.08(0.96–1.32) mmol/L vs. 1.32(1.16–1.57) mmol/L, *P* = 0.004] were lower in the EH group than in the control group. Level of TG was higher in the EH group than in the normal endometrium and EP group [1.89(1.08–2.92) mmol/L vs. 1.08(0.87–1.58) and 1.09(0.80–1.81) mmol/L, *P* = 0.025]. Hemoglobin level was lower in the EC group than in the other three groups [115.00(100.00-121.00) g/L vs. 132.00(126.00-140.00), 128.00(121.00-135.00), 129.00(113.50-138.75) g/L, *P* = 0.000].

**Table 2:** Comparison of clinical laboratory indexes among polycystic ovary syndrome patients with different endometrial status

Parameters	All patients (n = 234)	Normal endometrium(n = 98)	Endometrial polyp (n = 92)	Endometrial hyperplasia (n = 33)	Endometrial cancer (n = 11)	F/H	*P* value
PRL (ug/L)	11.54(8.27–17.95)	11.90(7.75-18.00)	11.25(8.25–17.58)	11.71(8.47–18.42)	11.64(8.43–18.52)	5.226	0.156
FSH (IU/L)	6.78(5.72–7.76)	6.60(5.46–7.76)	6.85(6.07–7.84)	6.71(4.73–7.39)	6.87(5.84–7.86)	3.168	0.366
LH (IU/L)	10.00(5.93–13.85)	9.77(6.18–12.09)	10.53(6.06–15.57)	8.71(5.01–16.13)	9.05(6.40-16.37)	1.924	0.588
E2 (ng/L)	50.00(36.00-72.50)	50.50(36.00-70.50)	47.50(33.00-72.75)	53.00(43.00–88.00)	52.00(38.00–79.00)	3.901	0.272
P (ug/L)	0.48(0.26–0.75)	0.40(0.26–0.67)	0.52(0.29–0.82)	0.39(0.23–0.75)	0.48(0.28–0.78)	3.404	0.333
T (nmol/L)	2.08(1.49–2.69)	2.17(1.58–2.77)	2.06(1.48–2.62)	1.77(1.27–2.44)	1.93(1.35–2.83)	1.880	0.598
DHEA (ug/dL)	295.45(192.03-447.68)	283.70(190.40-333.60)^a^	488.10(231.05-1301.98)	276.50(180.86-1256.79)	288.17(225.76-1252.76)	19.529	0.000
17α hydroxy progesterone(ng/ml)	1.09(0.81–1.62)	1.12(0.84–1.59)	1.10(0.75–1.75)	0.99(0.57–1.26)	1.04(0.96–1.54)	3.692	0.297
SHBG (nmol/L)	43.27(27.32–73.72)	44.68(28.82–73.24)^b^	49.04(29.38–81.67)	25.44(15.05–35.77)	46.05(26.41–63.49)	7.841	0.049
FAI	4.95(2.81–8.48)	4.71(2.60–8.06)	4.62(3.23–6.80)	6.91(4.89–14.42)	4.40(3.55-7.00)	5.936	0.115
AMH (ng/ml)	8.98(6.00-12.52)	9.12(5.52–12.63)	8.22(5.71–11.65)	9.66(6.64–15.37)	9.32(5.97–13.83)	0.726	0.696
GLU0 (mmol/L)	5.00(4.70–5.30)	5.00(4.75–5.30)	5.00(4.73–5.30)	5.05(4.61–5.80)	4.85(4.70–5.25)	0.494	0.920
GLU 2 h (mmol/L)	7.00(6.05-9.00)	7.05(6.20–8.50)	6.80(5.60–8.40)	7.70(6.28–10.83)	8.70(6.33–11.13)	4.291	0.232
INS 0 (mU/L)	11.23(7.46–18.38)	10.44(6.86–17.53)	11.51(8.25–19.06)	13.85(9.77–20.11)	11.60(4.90-24.41)	4.432	0.218
INS 2 h (mU/L)	99.31(58.53-174.98)	94.18(50.18-159.88)	88.56(61.99-182.37)	134.68(75.42-196.41)	107.60(30.32-237.17)	3.293	0.349
HOMA-IR	2.49(1.66–4.19)	2.33(1.51–4.03)	2.51(1.82–4.16)	3.44(1.74–4.88)	2.46(1.02–5.21)	3.585	0.310
TSH (mU/L)	1.71(1.18–2.55)	1.63(1.07–2.40)	1.95(1.48–2.83)	1.56(1.15–2.54)	2.83(2.83–2.83)	5.651	0.130
TC (mmol/L)	4.97(4.32–5.65)	5.16(4.41–5.74)	4.87(4.34–5.47)	4.95(4.29–5.95)	4.51(3.94–5.32)	3.439	0.329
TG (mmol/L)	1.19(0.85–1.81)	1.08(0.87–1.58)^b^	1.09(0.80–1.81)^b^	1.89(1.08–2.92)^a^	1.16(0.61–1.51)	9.326	0.025
HDL (mmol/L)	1.24(1.06–1.45)	1.32(1.16–1.57)^b^	1.22(1.06–1.41)	1.08(0.96–1.32)	1.08(0.90–1.34)	13.444	0.004
LDL (mmol/L)	3.13(2.69–3.63)	3.17(2.66–3.67)	3.08(2.69–3.59)	3.42(2.72–3.80)	2.85(2.52–3.31)	2.645	0.450
WBC (×10^9^ per L)	6.69(5.59–7.85)	6.76(5.61–8.50)	6.33(5.49–7.74)	7.15(5.87–7.84)	6.98(5.19–8.08)	2.408	0.492
RBC (×10^12^ per L)	4.55(4.27–4.78)	4.56(4.35–4.81)	4.50(4.28–4.77)	4.57(4.08–4.90)	4.63(4.08–4.92)	1.524	0.677
Hb (g/L)	128.50(121.00-136.00)	132.00(126.00-140.00)^ac^	128.00(121.00-135.00)^c^	129.00(113.50-138.75)^c^	115.00(100.00-121.00)^ab^	20.428	0.000
Prothrombin time(sec)	11.50(11.10–11.90)	11.80(11.20–11.90)	11.40(11.08–11.90)	11.40(11.00-12.10)	11.50(10.80–12.50)	2.586	0.460
Fibrinogen(g/L)	2.72(2.37–3.23)	2.81(2.43–3.25)	2.63(2.18–3.13)	2.94(2.53–3.43)	2.77(2.53–3.52)	5.573	0.134
APTT (sec)	26.72 ± 3.68	26.52 ± 3.12	27.25 ± 3.82^c^	26.46 ± 3.79	24.02 ± 3.45^a^	2.765	0.043
D-dimer(mg/L)	0.20(0.13–0.31)	0.21(0.14–0.41)	0.17(0.12–0.31)	0.20(0.17–0.28)	0.24(0.17–0.47)	2.962	0.398

*Data are medians (interquartile range) or means ± standard. a compared to endometrial polyp, p < 0.05; b compared to endometrial hyperplasia, p < 0.05; c compared to endometrial cancer, p < 0.05*

*Abbreviations: AMH = anti-müllerian hormone. APTT = activated partial thromboplastin time. DHEA = dehydroepiandrosterone. E2 = estradiol. FAI = Free androgen index. FSH = follicle-stimulating hormone. GLU = glucose. Hb = hemoglobin. HDL = high density lipoprotein. HOMA-IR = homeostasis model insulin resistance index. INS = insulin. LDL = low density lipoprotein. LH = luteinizing hormone. P = progesterone. PT = prothrombin time. PRL = prolactin. RBC = red blood cell count. SHBG = Sex hormone binding globulin. T = testosterone. TC = total cholesterol. TG = triglyceride. TSH = thyroid stimulating hormone. WBC = white blood cell*

**Fig. 1:**
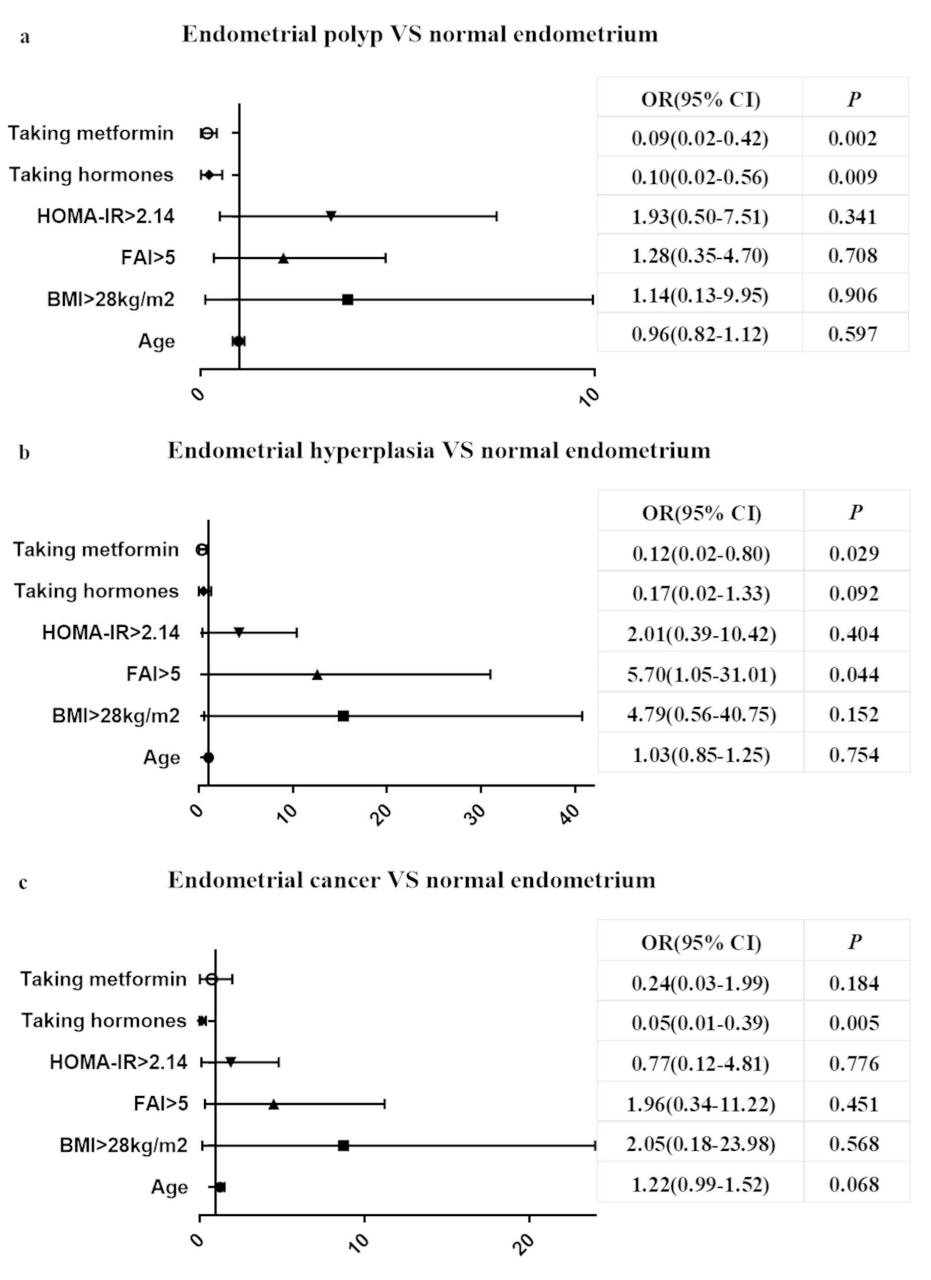
Multivariate analysis of independent risk factors associated with different endometrial status (**a** endometrial polyp VS normal endometrium, **b** endometrial hyperplasia VS normal endometrium, **c** endometrial cancer VS normal endometrium). OR, odds ratio; CI, confidence interval

In the multivariate logistic regression analysis, a significantly higher risk of EH was observed in patients with FAI > 5 (OR, 5.70; 95% CI, 1.05–31.01; *P* = 0.044). Taking metformin or hormones appeared to be protective factors against EP (OR, 0.09; 95% CI, 0.02–0.42; *P* = 0.002; OR, 0.10; 95% CI, 0.02–0.56; *P* = 0.009). Taking metformin appeared to be a protective factor against EH (OR, 0.12; 95% CI, 0.02–0.80; *P* = 0.029), while taking hormones appeared to be a protective factor against EC (OR, 0.05; 95% CI, 0.01–0.39; *P* = 0.005) Fig. 1.

## Discussion

In current study, we showed that PCOS women with EH have evidence of advanced age, obesity, prolonged menstrual cycle, decreased SHBG, and dyslipidemia, compared with PCOS women with normal endometrium. Meanwhile, taking metformin or hormones was a protective factor for endometrial lesions in PCOS patients. More importantly, we also revealed that the evident elevation of EH within the hyperandrogenic PCOS phenotype after statistical correction for differences in age, BMI and HOMA-IR, suggesting that hyperandrogenism, rather than obesity or insulin resistance, mainly contributes to EH.

PCOS is a complex multigenic disorder and women with PCOS suffer from several comorbidities. Prolonged endometrial proliferative phase is a typical feature of patients with PCOS. Studies have shown increased incidence of endometrial lesions in PCOS patients [2, 6–8]. A retrospective cohort study that analyzed endometrial pathology in 916 women demonstrated that patients with BMI > 30 developed endometrial lesions four times more often than those with normal weight [9]. Despite several endometrial abnormalities in women with PCOS were discovered, the clinical relevance of these findings still awaits future clarification. To date, no common screening protocols/recommendations for women with PCOS have been established.

EPs are one of the most common causes of abnormal uterine bleeding and a main reason of severe anemia [10]. There are very few studies on PCOS along with the occurrence of endometrial polyps. Our study found that there was no significant difference in T levels between the two groups, while the DHEA level in EP group was higher than that in control group, suggesting that DHEA levels may be associated with the occurrence of EPs. In the future, the relationship between PCOS patients with EPs and DHEA can be further explored by increasing the sample size.

Two extremely high-risk populations of EH are (i) obese peri/postmenopausal women, partly owing to peripheral aromatization of androgens to estrogens in adipose tissue, coupled with erratic anovulatory cycles and (ii) premenopausal patients with PCOS due to hyperandrogenic anovulation [11]. Our study showed that BMI of EH group was significantly higher than those of control group. Obesity is an important risk factor for complex EH in premenopausal women with abnormal uterine bleeding. Evidence suggests that adipocytes produce VEGF-mTOR signaling to stimulate endometrial cell proliferation, which leads to hyperplasia and cancer [12], and prolonged excessive estrogen exposure or lack of progesterone results in endometrial overgrowth and atypical endometrial hyperplasia [11]. The results of this study indicated that estrogen levels were not the main factor leading to PCOS EH, but further researches were needed. However, our study showed that compared with the control group, the level of SHBG decreased in EH group, and hyperandrogenism is an independent risk factor for EH. The reduction of SHBG can lead to an increased level of free androgen in the blood circulation. Elevated androgens may inhibit ovulation, while progesterone deficiency greatly increases the risk of EH. Researches are required to explore the relationship between hyperandrogenism and endometrial hyperplasia.

The relationship between PCOS and EC was first presented in 1949 [13]. Women of all ages with PCOS are at an increased risk of EC [14]. Chronic anovulation, obesity [15] and hyperinsulinemia are associated with both PCOS and EC [13]. In obese women, the conversion of estrogen to low-potency catechol estrogens is slow, resulting in relatively high levels of biologically active estrogen. Given that women with PCOS have been shown to have a three-fold increased risk of EC compared to women without PCOS [16], direct mitogenic effects [17], elevated bioavailable estrogen levels through a reduction in SHBG levels [18], hyperinsulinemia, and decreased apoptosis have been suggested as potential mechanisms. However, the specific molecular mechanisms of increasing EC risk in PCOS remain unclear.

Evidence had shown that diabetes mellitus increased the risk of endometrial cancer [19]. Hyperinsulinaemia is common finding prior the diabetes onset. IR is an important potential risk factor of EC. That IR induces high levels of insulin has direct and indirect effects for the development of EC. Women with PCOS and EC have an increased expression of genes (IGF1, IGFBP1 and PTEN) involved in the insulin signaling pathway in endometrial cells compared with control group [20]. However, IR does not appear to be a major factor in endometrial lesions in PCOS patients of reproductive age in our study. It is possibly because the PCOS patients we studied have a higher incidence of insulin resistance than the general population. Jamil et al. [21] found there were no differences in the incidence of metabolic syndrome and insulin resistance among different phenotypes of PCOS.

On one hand, our study suggests that both endometrial polyps and endometrial hyperplasia are related to elevated androgen, and there is no significant difference in IR between groups. However, treatment of PCOS with metformin can reduce the occurrence of endometrial lesions [22], possibly because treatment of IR can reduce androgen levels, promoting the recovery of menstruation [23], thereby reducing the occurrence of endometrial lesions. The pleiotropic effects of metformin on cellular energy metabolism as well as intercellular and hormone-based interactions make it a promising candidate for reprogramming of the cancer ecosystem [24] and a new adjunctive strategy of EC [25]. Animal experiments showed that metformin had increased efficacy against EC in obese versus lean mice and reversed the detrimental metabolic effects of obesity in the endometrial tumors [26].

Moreover, metformin has shown significant value in reversing EH in animal and human studies. As a fertility-sparing treatment in EH patients, metformin combined with megestrol acetate showed a higher early complete response rate compared with megestrol acetate alone [27]. Evidence suggested metformin alleviates the risk of EH in PCOS via the mTORC1/autophagy/apoptosis axis [28]. Hu et al. [29] found that metformin differentially modulated oestrogenic-stimulated protein expression of epithelial-mesenchymal transition in PCOS patients, which protected the endometrial function. Wang et al. [30] demonstrated that metformin might directly reverse impaired glycolysis and restore mitochondrial function in PCOS patients with EH.

On the other hand, combined oral contraceptives are beneficial to reduce the incidence of EP, EH and EC [31]. Progestogen (progesterone and progestin) can prevent or treat hyperplasia, atypical hyperplasia, and even well-differentiated EC. Progestins are the first choice for fertility preservation in young patients with grade 1 EC or atypical EH. Oguz et al. [32] found a progestogen with high anti-estrogenic activity may play an important preventive role in the development of EPs. Furthermore, the levonorgestrel-releasing intrauterine system can also alleviate heavy menstrual bleeding and anemia as well as EH and cancer, reduce the occurrence of endometrium lesion in users of tamoxifen [33]. Progestogens protect the endometrium from the proliferative effects of estrogens during menopausal hormone therapy [34]. The addition of metformin seems provide more benefits [35]. In EH patients without atypia, the endometrial reversal rate increased from 67 to 72% for oral progestins and from 81 to 94% for LNG-IUS, respectively. Injectable medroxyprogesterone acetate can also be considered as an alternative to LNG-IUS, with the endometrial reversal rate reaching 92% at 6 months [36].

Therefore, health education should be carried out to reduce fat and muscle mass and to decrease the risk of EH for obese PCOS patients. Occurrence of metabolic syndrome increases with age in PCOS patients and its incidence in non-obese patients is lower than that in obese patients [37]. Given the prevalence of overweight, obesity and insulin resistance, a relatively low reduction in weight (about 5%) can improve problems such as insulin resistance, high levels of androgens, reproductive system dysfunctions and infertility in these women [38]. We recommend monitoring blood pressure, serum lipids, serum glucose and other metabolic aspects of PCOS patients, and they are encouraged to control diet and exercise to keep fit.

There are some limitations in our study. Firstly, it is a retrospective case-control study, whose major problem is the small number of subjects in each group. Secondly, there is a potential selection bias since the study only included PCOS patients in one center. Thirdly, we did not consider the heterogeneity of PCOS and address differences between phenotypes. Well-designed prospective studies are needed to have a better understanding of the pathogenesis of endometrial lesions in PCOS patients.

## Conclusion

In conclusion, we have reported that age, obesity, prolonged menstrual cycle, decreased SHBG, and dyslipidemia may affect EH in PCOS, and hyperandrogenism may be an important cause of EH in such patients. More attention should be paid to the physical examination, endocrine, glucose and lipid metabolism screening of PCOS women. Oral contraceptives, progestogen and metformin are recommended for prevention and treatment of endometrial lesions in PCOS patients. However, additional prospective clinical studies with larger sample sizes should be performed to confirm our findings.

## Data Availability

The data that support the findings of this study are available from the corresponding author upon reasonable request, subject to institutional and ethical board approvals.

## Abbreviations

AMH: Anti-müllerian hormone
BMI: Body Mass Index
E2: Estradiol
EC: Endometrial cancer
EH: Endometrial hyperplasia
EP: Endometrial polyp
FAI: Free androgen index
FSH: Follicle-stimulating hormone
HDL: High-density lipoprotein
HOMA-IR: Homeostasis model assessment-estimated insulin resistance
LDL: Low-density lipoprotein
LH: Luteinizing hormone
OGTT: Oral glucose tolerance test
PCOS: Polycystic Ovary Syndrome
SHBG: Sex hormone binding globulin
T: Testosterone
TC: Total cholesterol
TG: Triglyceride
TSH: Thyroid stimulating hormone.
